# Data Association Methodology to Improve Spatial Predictions in Alternative Marketing Circuits in Ecuador

**DOI:** 10.1155/2018/6587049

**Published:** 2018-11-05

**Authors:** Washington R. Padilla, Jesús García

**Affiliations:** ^1^Salesian Polytechnic University of Quito-Ecuador Engineer Systems, Research Group Ideia Geoca, Quito, Ecuador; ^2^Carlos III University, Applied Artificial Intelligence Group, Madrid, Spain

## Abstract

This work proposes a methodology that reduces the error of future estimations in commercialization based on multivariate spatial prediction techniques (cokriging) considering the products with strong associations. It is based on the *Apriori* algorithm to find association rules in sales of agricultural products of local markets. Results show the improvement in spatial prediction accuracy after using the best association rules.

## 1. Introduction

Family farming is an economic and social sector provider of food in the world that guarantees processes of food security within a country. In Latin America and the Caribbean, family farming is the main source of agricultural and rural employment, comprising 80% of farms that represent around 60 million people. This type of productive model combines agriculture, livestock, forestry, fishing, aquaculture, and grazing within the same farm and provides on average between 27 and 67% of the total food production for each country in Latin America [1].

This type of family farming has certain characteristics:

- High presence of labor and family administration
- It is a diverse agriculture that allows self-sufficiency but also guarantees the feeding of other families through the surplus
- It is an agriculture that has limited access to productive resources such as land, water, and working capital compared to large-scale operations
- As an intangible heritage, family farming develops its own social and cultural dimension, which generates intergenerational links for the transfer of knowledge, traditions, and customs
- Generates social and community ties through the generation of cooperatives

Due to its productive and social characteristics, family farming is not a sector solely focused on production but also on the commercialization of products. However, the active participation of these farmers, focused on producing food for consumption in markets, is part of the sustainable and participatory development of the sector. In this sense, there are several limitations such as the geographical dispersion of the different farms of family farming, the production volumes of each family farm, and the limited capacity to meet quality standards established by marketing chains that demand access to markets. As indicated by Contreras et al., a lack of adequate coordination between the consumer and producer in the production and marketing system is not allowing adequately to respond to new demands or dissatisfaction of the consumer [2].

In response to this problem, local initiatives have emerged from family farmers to access markets such as the alternative marketing circuit (CIALCO). These spaces that propose the direct encounter between producers and consumers, in recent years, have acquired a high importance within the agendas of public policy for the development of family farming. This importance is linked to the local assessment of production through the promotion of a more local food consumption focused on the assessment of the agrobiodiversity of each territory [3]. These short marketing circuits are characterized as follows:

- Low or no presence of intermediation for the commercialization of products
- Generation of bonds of trust and closeness between producers and consumers
- Assessment of the temporality of production of each product.

This type of direct marketing can be presented through different strategies such as public purchase, fairs, or local markets for the sale of food, among other modalities. In Ecuador, these initiatives promoted by family farmers are supported by the Ministry of Agriculture and Livestock through the strengthening of local fairs to meet producers and consumers, the baskets of family farming, the export of family farming products, and the public purchase, among other initiatives. At the same time, this promotion has the objective of generating agricultural, environmental, and social policies that improve public policies aimed at responding according to the challenges and needs faced by family farmers, which make public action and its impacts effective, equitable, and sustained development of this sector. So far one of the constraints for the generation of public policies according to the needs of family farming is the scarcity of information on production, but also the data related to the income obtained by the marketing of their products [4].

The research objective is to generate a methodology to improve the prediction of commercialization of different agricultural products using geostationary spatial data mining techniques, using the existing data corresponding to the year 2014 that allows generating future scenarios for the evaluation of public policies that help the development of the family agricultural sector.

**Figure 1:**
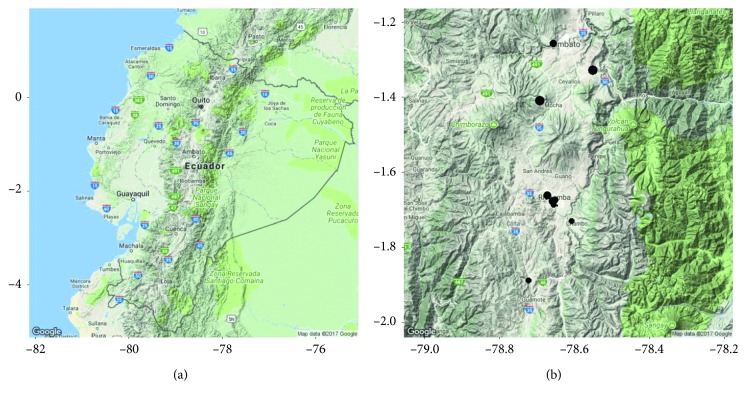
Geographic location: (a) the country Ecuador; (b) Tungurahua and Chimborazo.

The country Ecuador is crossed by the equatorial line, that means its territory is located both north and south of Latitude Zero (Figure 1(a)). At the south-central region are located the provinces of Tungurahua and Chimborazo (Figure 1(b)). From these two provinces, information has been collected on the sale of agricultural products in the so-called alternative marketing circuits (CIALCO).

The paper is organized in six sections, and it begins with a description of alternative marketing circuits, focuses on the main problem that is the lack of historical data that does not allow using statistical techniques, and presents the alternative use of algorithms used in data mining for the generation of future estimates in the commercialization of agricultural products generated by peasant families in specific places in the provinces of Tungurahua and Chimborazo in Ecuador.

In the second section, we present other works that use data mining techniques used in this research oriented to different domains establishing validity and probity of the algorithms used such as association rules, kriging, and cokriging, in addition to the relationship with topics oriented in the same line of research.

In Section 3, the theoretical description of the methods and data mining algorithms are presented, emphasizing their mathematical development, and the description of the information provided on which the different processes are applied is also presented in this section.

Section 4 describes the methodology proposed to improve the process of future estimates in the commercialization of products, a multivariable function is generated using the products resulting from the association rules, and it is verified if the errors in the prediction tend to decrease.

In Section 5, the proposed methodology is applied and, with the values obtained, the percentage of error reduction in the predicted values is calculated using algorithms for multivariable.

To conclude in the last section, using the percentage of improvement in future predictions, a tomato production scenario is presented graphically in the provinces of Tungurahua and Chimborazo in Ecuador, which allows to establish policies to improve the functionality in this type of circuits.

## 2. Related Work

This section overviews some relevant previous works related to the developed research, both in theoretical and practical aspects. A series of studies conducted in various fields of science try to use the rules of association as a criterion to establish future estimates, so we can see some works such as [5], whose authors analyze the stock of a supermarket, or [6], to predict admission decisions for students. In works like [7], a relationship between association rules and a fuzzy classification is established.

In [8], an explanation of the mathematical development of kriging and cokriging based on substitution models within the framework of optimization is made, [9] and they propose to improve the construction of the variogram using information of magnitude and direction applied to data of the National Network of the Geomagnetic Observatories of China.

In spite of researching specific works, there is very little documentation on the improvement in future estimation using association rules, as of 2016 the works that focus on this topic in a specific way [10] give the first guidelines in a process to establish the most consumed products and the best association rules that produce the first patterns with the highest consumption of family farming in Ecuador, and using rules of association, here we can see the generation of the first scenarios in the consumption of the products.

In the second work [11], it is the set of products obtained from the application of the Apriori algorithm, inside an improvement that oscillates in a value between twenty and thirty percent, the estimates of future sales using time series considering the half-squared error, and this work establishes scenarios regarding the periodicity of a product.

The third work [12] focuses on the estimation of the commercialization of products using their geographical location and their relationship as an influence in the improvement of consumption predictions based on the set of items resulting from the application of association rules.

The research developed in the area of statistical science has the concepts mature enough to deal with this type of approach with great solvency; however, in this particular case, there is not a sequence of data of several years that allow to use statistical techniques. Available data are limited to 2014, creating an appropriate scenario to test data mining techniques that allow to establish future estimates of product consumption, with these results it is expected to generate scenarios where the implementation of policies to improve alternative circuits can be evaluated by marketing.

## 3. Methods and Materials

This section presents in detail the theoretical basis of the data mining processes used, and a detail of the information on which the future estimates are made.

### 3.1. Association Rules and Apriori Algorithm

The first way to establish a relationship between products in this research is based on the number of times some products appear together in a sale transaction [13], and for this it is necessary to discretize the transactional data file, so that if a product is acquired, it is identified with a value T of true and F if it is not a part. This differentiation of products acquired allows to establish the minimum support that is known as the relationship between the number of times a product appears in a transaction with respect to the total of transactions made, and this process is repeated for a single item. Once the sets that meet for an item are established, we proceed to a similar calculation with two items and so on, identifying all sets that meet a preestablished minimum coverage, looking for the rules that meet a minimum of confidence, i.e., if the product appears in the antecedent of a rule, it has a minimum confidence level of appearing in the consequent of the rule.

The pseudocode is shown below.

#### 3.1.1. Pseudocode Algorithm Apriori [14]

**Step 1 .**

Generate all item sets *L* with a single element; this set is used to form a new set with two, three, or more elements all possible pairs which are taken as Sup equals minsup

**Step 2 .**

For every frequent item, set *L*′ is found:

  For each subset *J*, of *L*′
    Determine all association rules of the form:
    If *L*′-J⟶J
    Select those rules whose confidence is greater or equal than minconf

Repeat Step 1, including next element into *L*

One of the best known algorithms to search for association rules is the Apriori method [15]. It is based on two parameters support and confidence:

(i) The *support* of a rule is defined as the number of instances that the rule correctly predicts:
  (1) $$\begin{array}{c} \sup_{a}\left(x\right)=\left|x\right|,\;\;\sup_{r}\left(x\right)=\frac{\left|x\right|}{\left|D\right|},\text{ being }D\text{ the total set of transactions},\\ \sup_{a}\left(A\Rightarrow B\right)=\sup_{a}\left(A\cap B\right). \end{array}$$
(ii) The *confidence* indicates the percentage of times that a rule is met among the instances selected by the antecedent A:
  (2) $$\begin{array}{c} \mathrm{conf}\left(A\Rightarrow B\right)=\frac{\sup_{a}\left(A\Rightarrow B\right)}{\sup_{a}\left(A\right)}=\frac{\left|A\cap B\right|}{\left|A\right|}. \end{array}$$

### 3.2. Spatial Estimation

As mentioned in [16], “in the geographical space everything is related to everything, but the closest spaces are more related to each other”. Geostatistics use the concept of a random function to find nondeterministic values on a region *D*, and if *x* crosses the region, a series of random variables are obtained, defined as

(3) $$\begin{array}{c} Z=\left\{Z\left(x\right),\;\;x\;\varepsilon D\right\}, \end{array}$$

which constitutes a random function on domain *D*.

To simplify the feature of the random function, we consider some descriptive parameters or moments that summarize the information, the expectation, or first-order moment *m*(*x*)=*E*[*Z*(*x*)], represent the average around which the values taken by the realizations of the random function are distributed, and the variance is calculated as follows:

(4) $$\begin{array}{c} \sigma^{2}\left(x\right)=\mathrm{var}\left[Z\left(x\right)\right]=E\left\{{\left[Z\left(x\right)-m\left(x\right)\right]}^{2}\right\}=E\left[Z\left(x\right)2\right]-m{\left(x\right)}^{2}, \end{array}$$

and the variance and its square root called standard deviation constitute measures of dispersion of *Z*(*x*) around its mean value; the covariance centered between two random variables is given by the relationship

(5) $$\begin{array}{c} C\left(x_{1},x_{2}\right)=E\left[Z\left(x_{1}\right)Z\left(x_{2}\right)\right]-m\left(x_{1}\right)m\left(x_{2}\right), \end{array}$$

and gives us an elementary vision of the interaction that exists between *Z*(*x*_1_) and *Z*(*x*_2_), and the semivariogram, defined between the two random variables, is given by the expression

(6) $$\begin{array}{c} \gamma \left(x_{1},x_{2}\right)=\frac{1}{2}var\left[Z\left(x_{1}\right)-Z\left(x_{2}\right)\right], \end{array}$$

and it reflects the way in which a point has influence on another point at different distances. The variogram is equal to the variance minus the covariance:

(7) $$\begin{array}{c} \gamma \left(h\right)=C\left(0\right)-C\left(h\right). \end{array}$$

#### 3.2.1. Experimental and Modeling Variogram

If we consider the *z* regionalized variable known in *n* sites {*x*_1_,…, *x*_*n*_}, the estimator of the experimental variogram for a separation vector *h*, it is defined as follows:

(8) $$\begin{array}{c} \gamma \left(h\right)=\frac{1}{2}E\left\{{\left[Z\left(x+h\right)-Z\left(x\right)\right]}^{2}\right\}. \end{array}$$

An experimental variogram cannot be used because it is defined only for certain distances and directions, to interpret the spatial continuity of the study variable, and a theoretical model should be adjusted around the experimental variogram.

A variogram, *γ*(*h*), is isotropic if it is identical in all directions of the space and if it does not depend on the orientation of the vector *h* but only on its magnitude |*h*|; otherwise, there is anisotropy in its distribution [17].

In general, the modeled variogram grows from the origin and stabilizes at a distance *a*, around a plateau; the two random variables *Z*(*x*) and*Z*(*x*+*h*) are correlated if the length of the separation vector *h* is less than the distance *a*, called the reach or zone of influence, beyond |*h*|=*a*; and the variogram is constant and equal to its plateau.

A spherical variogram of reach *a* and plateau *C* is defined as

(9) $$\begin{array}{c} \gamma \left(h\right)=\left|\begin{array}{cc} C\left\{\frac{3}{2}\frac{\left|h\right|}{a}-\frac{1}{2}{\left(\frac{\left|h\right|}{a}\right)}^{3}\right\}si, & \left|h\right|\leq a,\\ C, & \mathrm{otherwise}. \end{array}\right. \end{array}$$

In processes involving geostatistics, the spatial correlation is modeled by the variogram, and this process is generated by a random function *Z*(*s*) composed by the mean (*m*) and the residue e(s): *Z*(*s*)=*m*+*e*(*s*), with an average constant *E*(*Z*(*s*))=*m*, and the variogram defined as *γ*(*h*)=1/2*E*(*Z*(*s*) − *Z*(*s*+*h*))^2^. The variance of *Z* is constant, and the correlation of *Z* does not depend on the location *s* but only on the separation distance *h*. Then, we can form multiple pairs {*Z*(*s*_*i*_), *Z*(*s*_*j*_)}, that have identical separation vector *h* = *s*_*i*_ − *s*_*j*_, and we estimate the correlation between them [18, 19]. An experimental variogram *γ*(*h*) is isotropic if it is identical in all directions of space; otherwise, there is anisotropy.

If we assume the entropy is in the independent direction of the semivariance, we replace the vector *h* with the magnitude ‖*h*‖. Under this assumption, the variogram can be estimated for *N*(*h*) as a simple pair of data *Z*(*s*_*i*_), *Z*(*s*_*i*_+*h*). For some distances (intervals), $\bar{h_{j}}$ is defined as

(10) $$\begin{array}{c} \hat{\gamma }\left(\bar{h_{j}}\right)=\frac{1}{2N_{\left(h\right)}}\overset{N_{h}}{\underset{i=1}{\sum }}{\left(Z\left(s_{i}\right)-Z\left(s_{i}+h\right)\right)}^{2},\;\forall h\in \bar{h_{j}}, \end{array}$$

and this estimate is called a simple variogram.

The experimental variogram [20, 21] measures the average dissimilarity between two data as a function of their separation, often presents slope changes, which indicate a change in spatial continuity from certain distances, and the variogram can be modeled as the sum of several elementary models called models nested or nested structures [22]

(11) $$\begin{array}{c} \gamma \left(h\right)=\gamma_{1}\left(h\right)+\gamma_{2}\left(h\right)+\ldots +\gamma_{s}\left(h\right). \end{array}$$

The adjustment to a model is not done considering only the experimental variogram, but it must consider all the available information on the regionalized variable, and a more detailed explanation can be found in [23].

#### 3.2.2. Estimation with Kriging

The kriging method in this case is considered as a linear prediction with unbiased linear estimator, and there are some types of kriging depending on the average of the known population. These types are ordinary and simple, and for this study, we are interested in the ordinary type.

The regionalized variable is the obtaining of the stationary *Z* random function that fulfills

(12) $$\begin{array}{c} \left\{\begin{array}{cc} \forall x\in V, & E\left[Z\left(x\right)\right]=m\text{ unknown,}\\ \forall x,x+h\in V, & cov\left[Z\left(x+h\right),Z\left(x\right)\right]=C\left(h\right), \end{array}\right. \end{array}$$

where *V* is the neighborhood considered in the kriging process. The following conditions are considered:

(i) Linearity:
  (13) $$\begin{array}{c} Z^{*}\left(x_{0}\right)=a+\overset{n}{\underset{\alpha =1}{\sum }}\lambda_{\alpha }Z\left(x_{\alpha }\right). \end{array}$$
  where **x**_0_ is the place where an estimate is established, {*x*_*α*_, *α*=1,…, *n*} are the sites with known data, and {*λ*_*α*_, *α*=1,…, *n*} are the weights that together with “*a*” they are the unknowns.
(ii) *Unbiased* estimation constraint: it is expressed that the expectation of estimation error must be zero:
  (14) $$\begin{array}{c} E\left[Z^{*}\left(x_{0}\right)-Z\left(x_{0}\right)\right]=0\\ =a+\overset{n}{\underset{\alpha =1}{\sum }}\lambda_{\alpha }E\left[Z\left(x_{\alpha }\right)\right]-E\left[Z\left(x_{0}\right)\right]=a+m\left(\overset{n}{\underset{\alpha =1}{\sum }}\lambda_{\alpha }-1\right). \end{array}$$
(iii) Minimum variance: find weights that minimize the variance of the estimation error:
  (15) $$\begin{array}{c} \mathrm{var}\left[Z^{*}\left(x_{0}\right)-Z\left(x_{0}\right)\right]\text{it }\mathrm{is}\text{ minimal}\\ =\overset{n}{\underset{\alpha =1}{\sum }}\overset{n}{\underset{\beta =1}{\sum }}\lambda_{\alpha }\lambda_{\beta }C\left(x_{\alpha }-x_{\beta }\right)+C\left(0\right)-2\overset{n}{\underset{\alpha =1}{\sum }}\lambda_{\alpha }C\left(x_{\alpha }-x_{\beta }\right). \end{array}$$

Being the variogram, a tool equivalent to the covariance from the relationship,

(16) $$\begin{array}{c} \gamma \left(h\right)=C\left(0\right)-C\left(h\right). \end{array}$$

The calculation of kriging is done as follows:

(17) $$\begin{array}{c} \left\{\begin{array}{c} \overset{n}{\underset{\beta =1}{\sum }}\lambda_{\beta }\gamma \left(x_{\alpha }-x_{\beta }\right)-\mu =\gamma \left(x_{\alpha }-x_{0}\right)\;\forall \alpha =1\ldots n,\\ \overset{n}{\underset{\alpha =1}{\sum }}\lambda_{\alpha }=1, \end{array}\right.\\ \left(\begin{array}{ccc} \gamma \left(x_{1}-x_{1}\right) & \cdots \gamma \left(x_{1}-x_{n}\right) & 1\\ \vdots & \ddots & \vdots \\ \gamma \left(x_{n}-x_{1}\right) & \ldots \gamma \left(x_{n}-x_{n}\right) & 1\\ 1 & 1 & 0 \end{array}\right)\left(\begin{array}{c} \lambda_{1}\\ \vdots \\ \lambda_{n}\\ -\mu \end{array}\right)=\left(\begin{array}{c} \gamma \left(x_{1}-x_{0}\right)\\ \vdots \\ \gamma \left(x_{n}-x_{0}\right)\\ 1 \end{array}\right). \end{array}$$

#### 3.2.3. Multivariate Prediction: Cokriging

In this case, multiple spatial variables are analyzed together to build the prediction model. The first step is modeling a multivariable variogram, and the main tool for estimating semivariances between different variables is the crossed variogram, defined as follows:

(18) $$\begin{array}{c} \gamma_{ij}\left(h\right)=\frac{1}{2}E\left[\left(Z_{i}\left(s\right)-Z_{i}\left(s+h\right)\right)\left(Z_{j}\left(s\right)-Z_{j}\left(s+h\right)\right)\right]. \end{array}$$

Two variables can have cross correlation, which means that the variables not only exhibit autocorrelation but that the spatial variability of a variable A is correlated with variable B, and vice versa. This can be extended to multiple variables; the measurements are taken in a limited set of locations, and the interpolation can be made to an unlimited number of locations. The cokriging seeks to estimate the value of a variable considering the data of this variable and other correlated variables, for this uses the following relationships [24, 25].

The crossed variogram between two variables *Z*_1_ and *Z*_2_ is defined as follows:

(19) $$\begin{array}{c} \gamma_{12}\left(h\right)=\frac{1}{2}\mathrm{cov}\left\{Z_{1}\left(x+h\right)-Z_{1}\left(x\right),Z_{2}\left(x+h\right)-Z_{2}\left(x\right)\right\}, \end{array}$$

and can be computed from the available data:

(20) $$\begin{array}{c} {\hat{\gamma }}_{12}\left(h\right)=\frac{1}{2\left|N\left(h\right)\right|}\underset{N\left(h\right)}{\sum }\left[Z_{1}\left(x_{\alpha }\right)-Z_{1}\left(x_{\beta }\right)\right]\left[Z_{2}\left(x_{\alpha }\right)-Z_{2}\left(x_{\beta }\right)\right], \end{array}$$

where *N*(*h*)={*α*, *β*, such that *x*_*α*_ − *x*_*β*_=*h*}, being both variables *z*_1_ and *z*_2_ measure in *x*_*α*_ and *x*_*β*_.

### 3.3. Materials

The analysis is based on information from 2014, provided by the General Coordination Network Marketing Ministry of Agriculture and Livestock of Ecuador. It contains the weekly performance of sales of agricultural products made by small farmers located in Ecuador's central highlands specifically the provinces of Tungurahua and Chimborazo. The available data contains information about the number and volume of sales of products such as vegetables, legumes, meat, dairy, fruits, tubers, and processed products, finding an average of 1,200 items per month divided on a weekly basis.

The elected products that have greater relevance in relation to information in the universe to be part of the research consists of thirty products, indicated in Table 1 (it contains the names in English, scientific name, and Spanish, the original language of the study). Further details of this dataset can be appreciated in the initial part of the investigation [26].

The available record contains the products that are part of the marketing, the value of sales, date, and fair to which each transaction belongs (Table 2).

On the one hand, the first data sheet contains all the recorded transactions, organized in packages named “canastas” (baskets), each one representing a sale of certain products, containing the products present in each purchase, and implicitly also contains the spatial geolocalization of the operation (the location of the fair) and the time stamp (date) of operation. As shown in Table 3, the transactions have the dates and, for each of the 30 products, the label with character “s” denotes it was present in transaction, and otherwise “No”. This table contains 550 transactions recorded along all months in the year 2014. This table containing binary attributes was the base for the association analysis performed in the first place.

On the other hand, Table 3 contains the sales value reported for each product aggregated in weeks and locations. It contains the numerical attributes reflecting the weekly variation of sales, with a blank space when there is no registered value. This second table, containing the sales information of 48 weeks with a total of 1260, was based to carry out the prediction analysis.

## 4. Proposed Methodology

The proposed methodology to improve the prediction of commercialization of products consists in searching the set of elements with the highest degree of associativity in commercialization. It is used to reduce the error in the spatial estimate of commercialization of agricultural products. It consists in the following steps:

- Establish a baseline with future estimated values for the marketing of agricultural products, using the deterministic method IDW (inverse distance weight)
- Establish the set of associated products (using the *Apriori* algorithm of association rules)
- For the (unique “u”) selected product,
  - Establish the experimental variogram model and the theoretical variogram that best suits the existing data (the adjusted variogram)
  - Calculate the estimate of the behavior of a future product based on the prediction (kriging)
  - Carry out the cross validation to estimate the error of the waste (CVu residuals)
- For the set of products associated with the highest transaction ratio (multivariable “mv”)
  - Verify the correlation between the selected elements
  - Repeat steps 3.3.1 to 3.3.2 for the multivariable set
  - Carry out cross validation cokriging (CVmv residuals)
- Compare the residual values obtained in the two cases (Cvu and CVmv) with the future estimate values of IDW

Detail of implementation and results obtained from the proposed methodology are done in the following section.

## 5. Results and Discussion

### 5.1. Experimental Analysis

#### 5.1.1. Data Processing

**Figure 2:**
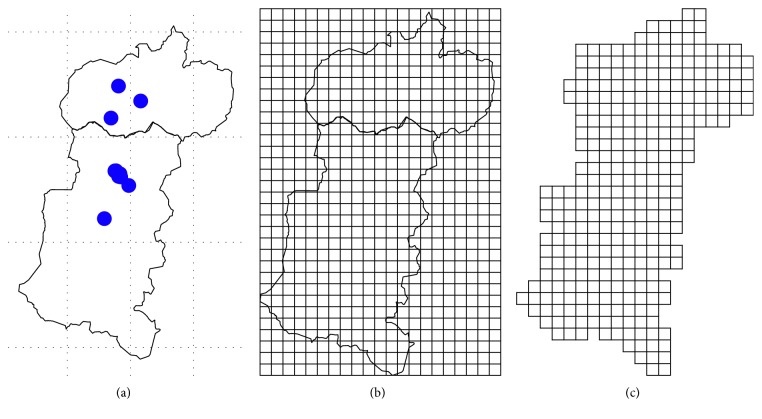
Prediction mesh: (a) fairs location; (b) mesh; (c) prediction area.

The proposed methodology has been applied to the marketing information of agricultural products provided by the Ministry of Agriculture of Ecuador, of the result collected from the different fairs located in Tungurahua and Chimborazo, with the data of sale of products of the month of July the year 2014 (Figure 2(a)).

To implement, the proposed methodology, we use the mathematical algorithms found in the R language, and the libraries used are SpatialPoints (sp), ggmap, tmap, ggplot2, GADMTools, rgeos, gdalUtils, gstat, geoR, proj4, crs, raster, maps, readr, in version 1.0.143 [27–30], and Weka 3.7 [31], and the generation of association rules is carried out.

The first activity is centered in the creation of the grid or mesh [32, 33] to determine the prediction area, and a dimension structure is defined with parameters: cellcentre.offset *x* = −79.1085, *y* = −2.531218, cellsize *x* = 0.05, *y* = 0.05, cells.dim *x* = 21; *y* = 32. In the sector of the equatorial line one degree of length equals 111.32 km, the distance occupied in length by the two provinces *x*_min_ = −79.133499 and *x*_max_ = −78.0834991 is 1.049 degrees, the equivalent to 116 km, for the conformation of the grid (spgridtc), and the distance between cells is 5.84 km (Figure 2(b)).

#### 5.1.2. Search for Association Rules

To find association rules, information must be quantized, so you can identify whether an agricultural product is part of the procurement process.

If part of the transaction is the label with the character “T”, otherwise “F” for all months of 2014, to optimize the process of searching for the best value association rules is replaced with “F” by the symbol “?”.

The Apriori algorithm for association rules is applied to a set of 550 transactions, with minimum support parameters equal to 0.4 (220 occurrences) and a confidence of 0.8. The resulting set is

**Figure 3:**
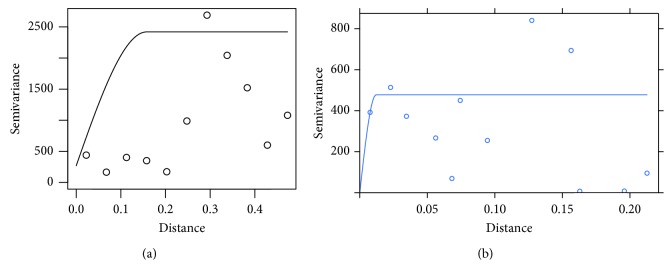
Tomato variogram: (a) variogram model; (b) adjusted variogram

**Figure 4:**
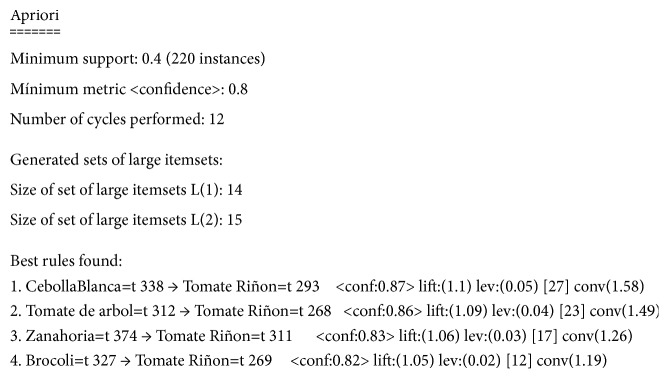
Association rules.

- Each time a white onion transaction is made, a tomato transaction is performed with a confidence of 87%, tamarillo (86%), carrot (83%), and broccoli (82%) (Figure 3), and each one of these elements generates a rule of association with the tomato, which constitutes the set of multivariable.
- The product with the highest commercial ratio of the study sample is tomato.
- The set of greater associativity is structured as A = {Tomato, White Onion, Tamarillo, Carrot, Broccoli} Figure 4.

With the set of best association rules, the source of data is generated on which the different estimation processes are carried out in the future, and this file is called Fjespacial Table 4.

#### 5.1.3. Baseline Analysis (IDW)

**Figure 5:**
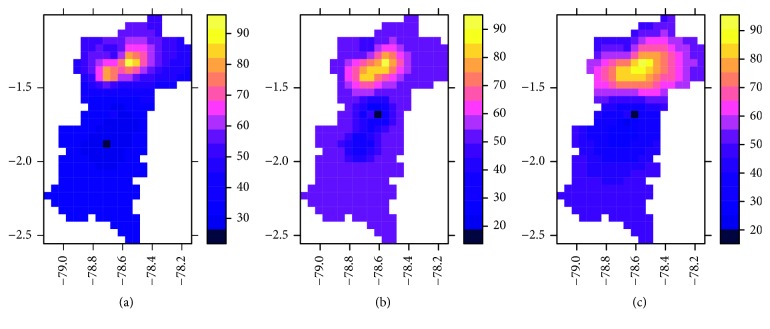
Tomato sales estimate: (a) IDW; (b) kriging; (c) cokriging.

In order to establish a baseline of analysis, the deterministic method inverse distance weighting (IDW) is used to calculate a first estimate in the future using the set of products with the greatest associativity such as broccoli, white onion, tomato, tamarillo, and carrot established in Section 5.2. The prediction of consumption for a single variable, idw ((TOMATE)∼1, FJespacial, spgridtc), where the variable to predict is the tomato, Fjespacial contains the values of sales, and spgridtc is called the grid or area where the prediction is made (Figure 5(a)).

### 5.2. Spatial Data Analysis

The data used correspond to the commercialization of the input denominated tomato of the month of July 2014, in the provinces of Tungurahua and Chimborazo, this file is converted to a spatial type, transforming the location data *x* and y into geographic coordinates [17, 34–36], that represent the latitude and length of each of the alternative circuits of commercialization type fairs that act in the study, and the fourth column corresponds to the values of the behavior of commercialization of tomato.

#### 5.2.1. Theoretical and Experimental Variogram Models

The distance between the points that identify the fairs is expressed in tenths of a degree, and between each jump, there is a distribution of two fairs.

The model variogram (m), of the spherical type with a range of 0.157, is where the spatially correlated points are found, with a plateau equal to 2151 and distance 0,473.

In Figure 3, the adjusted variogram can be observed using the experimental variogram and the model variogram.

#### 5.2.2. Kriging

Using the continuous function of the adjusted variogram, the tomato consumption prediction values are obtained based on distance and spatial correlation in the following way:

krige (TOMATE∼1, FJespacial, spgridtc, model = *m*), ∼1 defines a single constant predictor.

Based on the ordinary kriging method that is considered the best unbiased linear estimator type, the values found in the interpolation vary especially in two foci on which the predictions are generated.

The values closest to the points of information are more influenced than those that are far away (Figure 5(b)).

#### 5.2.3. Spatial Prediction Based on Associated Products

Because of the interrelation of products found with the *Apriori* algorithm, a set of associated products in the commercialization with the highest incidence in the process was identified. The five products resulting from association rules is A = {Tomato, Broccoli, White Onion, Tamarillo, Carrot}.

**Figure 6:**
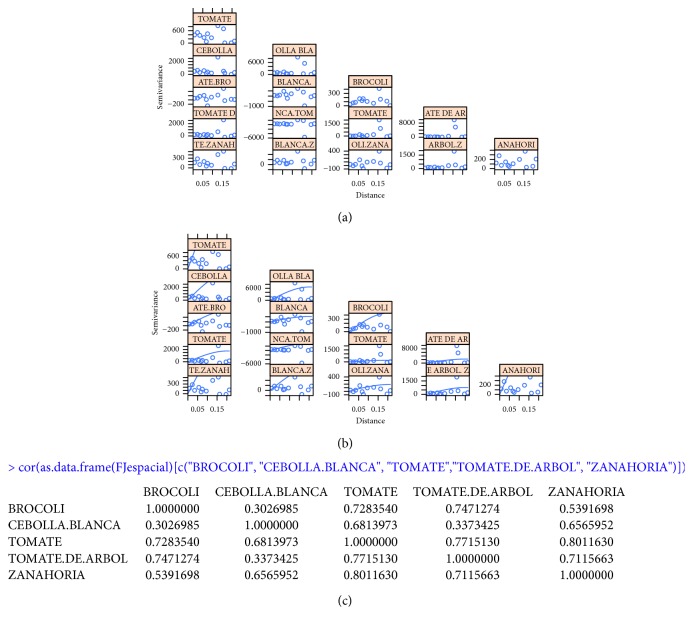
Multivariate correlation and variogram

The correlation between the elements of set A was verified, and the model variogram with each element will be generated, as can be seen in Figure 6.

At this point, the linear model of coregionalization is adjusted to a variogram of multivariable samples using the products.

#### 5.2.4. Cokriging

In the same way as made for the tomato variable, we proceed to estimate the future sales of the target variable (tomato), with an extended model integrating all the associated products, as shown in Figure 5(c). The variable *g* represents a function with all the products resulting from the added association rules for which the new variogram is calculated, vmra <- variogram (*g*).

The adjusted variogram is obtained from the interaction between the variogram of the function *g* (multivariable) and the model *m* of a single variable, vm.fit <- fit.lmc (vmra, *g*, and m).

The multivariate prediction is derived from the relation xt <- predict (vm.fit and spgridtc). A summary of the three cases of prediction of future consumption is presented in Figure 5.

### 5.3. Discussion

To perform the assessment of the prediction model, the cross validation divides the data into two sets: the modeling subset is used by the model variogram to estimate the coefficients, ant then, kriging is applied in the locations of the validation set, so that validation measures are compared with their predictions.

The procedure known as leave-one-out cross validation (LOOCV) was applied, and it performs as many iterations as data (*N*) has the set, using *N*−1 data to train the model and the data left for testing, being the result the arithmetic mean of the *N* error results obtained *E*=1/*N*∑_*i*=1_^*n*^ *e*_*i*_.

Cross validation usually gives a pessimistic estimate of performance (bias), since most models would improve if the training set would be bigger. For this reason, LOOCV has the lowest bias since the training set contains the whole dataset except one datum. On the other hand, some authors point out that the error estimated by LOOCV may have greater variance than *k*-fold cross validation, with *k*<<*n*, since the size of datasets is higher and estimation smoother. However, this is open to discussion, as indicated [37], since *k*-fold cross-validation produces dependent test errors, and their correlations cannot be estimated unbiasedly. As indicated, in [38], in learning problems employing models with moderate/low instability (as linear regression problems), LOOCV often has lower variability both in bias and variance.

In any case, for situations with small datasets the variance in fitting the model tends to be higher, implying that *k*-fold cross-validation is likely to have a high variance (as well as a higher bias) with respect to LOOCV. This is why LOOCV is often the best choice with limited amounts of available data, as the case study in this work, in order to get the maximal use of data to compare the performance of alternative learning structures.

The estimation error (difference between the estimated value and the true value) is calculated in each site with data, and a statistical analysis is made of the errors committed in all data sites.

**Figure 7:**
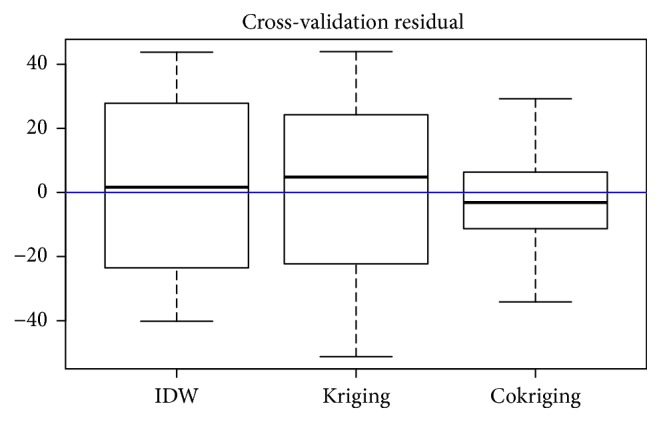
Comparative cross validation.

The results obtained from performing the cross validation for each method chosen for this study indicate that when comparing the residual values of the predictions, the IDW and kriging method have similar prediction values while the cokriging process (multivariate) presents a improvement for its smaller amplitude in the results (Figure 7). As can be seen in the cash flow diagrams, for all the estimation processes of future sales, the values are located in a range between −20 and 30.

**Figure 8:**
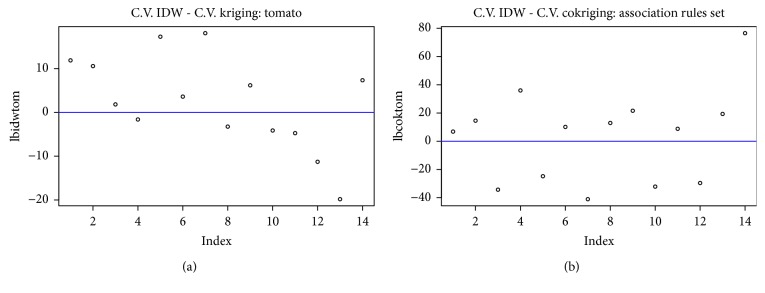
Cross validation relations.

Figure 8 shows the result of subtracting the residual values between the prediction of (1) IDW/kriging method (left frame) and (2) IDW/cokriging (right frame).

The first frame represents the set of values obtained from performing the cross validation of estimated values for the methods of future estimation residual between IDW and ordinary kriging (single variable), and the positive values are eight and the negative ones six.

In the second part, the residual difference of the cross validation between the IDW method and the ordinary cokriging (multivariable) is calculated, establishing nine positive and five negative values.

Positive samples indicate that the residual value of the multivariable function is smaller.

## 6. Conclusions

This research is focused on a target area for analysis located in Tungurahua and Chimborazo provinces of Ecuador, where there are fourteen alternative marketing circuits called fairs. These locations were used to create the grid of future sales estimates, and the analysis of sales transactions containing agricultural products generated the set of strongly associated products, based on the Apriori algorithm. As result, the set of associated products with support parameters = 0.4 and confidence greater than 0.8 are, in order, the following: white onion (0.87), tamarillo (0.86), carrot (0.83), and broccoli (0.82), and each of these products is associated with the sale of tomato.

**Figure 9:**
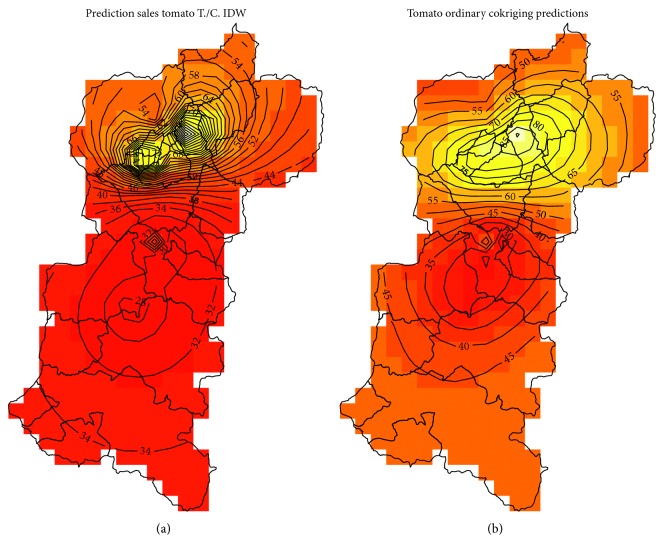
Results for scenarios.

Using the IDW process as baseline for comparison, the leave-one-out cross validation of predictions was done to compare with geostatistical techniques based on the variogram to generate interpolations of product sales in the target area. Based on the functions of kriging, the sales values of products were established according to their spatial locations and influences of close neighbors. Finally, a multivariable set of products for predictions was established resulting from association rules with greatest associativity (tomato, broccoli, tamarillo, white onion, and carrot). Based on this multivariable set, the prediction values are calculated using the same procedure described in the first and second stages. With this improvement in the sales prediction process, it would be possible to establish scenarios to generate consumption maps (Figure 9) that can be supplied with a better production process and its subsequent commercialization which is reflected in a better level of economic income for the farmer families.

Finally, the products resulting from applying the Apriori algorithm with the greatest associativity are tomato, broccoli, tamarillo, white onion, and carrot.

Based on this multivariable set, the prediction values are calculated using the same procedure described in the first and second stages.

The residual value of the IDW prediction minus kriging prediction delivers eight positive values and six negative values. In the same way, it is calculated for the IDW minus cokriging process, and in this case, nine positive and five negative values are obtained.

In the process of cokriging (multivariable), there are a greater number of cases with positive differences that shows that this process using the set of association rules as multivariable has a 16% improvement when establishing future sales estimate.

The proposed methodology is to find a set based on association rules to establish the multivariable process, and this research has shown acceptable improvement in prediction values. With this improvement in the sales prediction process, it is possible to establish scenarios to generate consumption maps (Figure 9) that can be supplied with a better production process and its subsequent commercialization which is reflected in a better level of economic income for the family farmer.

Finally, it should be emphasized that a methodology is established based on the use of association rules that allow future estimates to be improved using cokriging (multivariable) processes.

Taking into account that in order to use a conventional statistical process, there is not enough data available to establish a distribution and an estimate of future prediction, and it is considered that the proposed technique and methodology are useful for initial cases of study, have a limited amount of data, especially to create a baseline, as more annual data series are obtained which can be compared between the values obtained using data mining techniques contrasted with values of traditional statistical techniques.

## Figures and Tables

**Table 1:** Set of selected agricultural products.

Spanish name	Scientific name	English name
Acelga	*Beta vulgaris* var. cicla	Chard
Ajo	*Allium sativum*	Garlic
Arveja	*Pisum sativum*	Vetch
Babaco	*Carica pentagona*	Babaco
Brócoli	*Brassica oleracea* italica	Broccoli
Cebolla blanca	*Allium fistulosum*	White onion
Cebolla paiteña	*Allium fistulosum*	Onion
Choclo	*Zea mays*	Corn
Col	*Brassica oleracea*	Cabbage
Col verde	*Brassica oleracea* var. sabellica	Green cabbage
Coliflor	*Brassica oleracea* var. botrytis	Cauliflower
Espinaca	*Spinacia oleracea*	Spinach
Frejol	*Phaseolus vulgaris*	Frejol
Frutilla	*Fragaria*	Strawberry
Habas	*Vicia faba*	Broad beans
Hierbas	*Coriandrum sativum, Petroselinum crispum*	Weeds
Lechuga	*Lactuca sativa*	Lettuce
Melloco	*Ullucus tuberosus*	Melloco
Nabo	*Brassica rapa*	Turnip
Papas	*Solanum tuberosum*	Potatoes
Pepinillo	*Cucumis sativus*	Pickle
Pepino	*Cucumis sativus*	Cucumber
Pimiento	*Capsicum annuum*	Pepper
Rábano	*Raphanus sativus*	Radish
Remolacha	*Beta vulgaris*	Beet
Tomate de árbol	*Solanum betaceum*	Tamarillo
Tomate riñón	*Solanum lycopersicum*	Tomato
Vainita	*Phaseolus vulgaris* L	Vainita
Zanahoria	*Daucus carota*	Carrot
Zapallo	*Cucurbita máxima*	Zapallo

**Table 2:** Transactions with products contained in each sale (“canasta”).

	10/5/2014	10/5/2014	10/5/2014	10/5/2014	10/5/2014	10/5/2014	10/5/2014	10/5/2014	10/5/2014	10/5/2014	10/5/2014
Acelga	s	s	No	No	s	No	s	No	s	No	s
Ajo	No	No	No	s	No	s	s	s	No	No	No
Arveja	No	s	No	No	s	s	s	No	No	s	No
Babacos	No	No	No	No	No	No	No	s	No	No	No
Brócoli	s	s	No	s	No	s	s	s	s	No	No
Cebolla blanca	No	No	s	s	s	No	s	s	s	s	s
Cebolla paiteña	s	No	s	No	s	No	No	No	s	s	No
Choclo	s	s	s	No	s	s	s	No	No	s	No
Col	s	No	s	s	s	s	s	No	s	No	s
Coles Verde	No	No	No	No	No	No	No	No	No	No	No
Coliflor	No	No	No	s	No	s	No	No	No	No	No
Espinaca	No	No	No	No	No	No	No	No	No	No	No
Frejol	No	s	No	s	s	s	No	No	No	s	No
Frutilla	No	No	No	s	No	No	s	s	No	s	No
Habas	No	No	No	s	No	s	s	s	No	s	s
Hierbas	s	No	No	No	s	No	No	No	No	s	No
Lechuga	s	No	No	s	s	s	s	No	s	No	No
Melloco	No	s	No	No	No	No	s	No	No	No	No
Nabo	No	No	No	No	No	No	No	No	No	No	No
Papas	No	s	s	s	s	s	s	No	s	No	No
Pepinillo	No	No	s	s	No	No	No	No	s	s	No
Pepino	No	No	No	No	No	No	s	No	No	No	No
Pimiento	No	s	s	s	s	s	s	s	No	s	No
Rabano	No	No	s	s	s	No	No	s	No	No	s
Remolacha	No	No	No	s	No	No	s	No	s	No	No

**Table 3:** Value sale products.

Fecha	Acelga	Ajo	Arveja	Babacos	Brócoli	Cebolla blanca	Cebolla colorada	Cebolla paiteña	Choclo	Col	Col verde	Coliflor	Espinaca	Frejol	Frutilla	Habas	Hierbas	Lechuga	Melloco	Nabo	Papas	Pepinillo	Pepino	Pimiento	Rabano	Remolacha	Tomate de arbol	Tomate riñon	Vainita	Zanahoria	Zapallo
1/5/2014	13.75	21	82	9	32.8	44.5	26	41	56	22.5	8	11	4.25	5	38	84.5	23.5	18.25	26	3	180	16.5	12	17	6.5	3.5	42	121	2.5	29	8.5
1/12/2014	14.75	21	65	9	30.3	51.25	26	52	56	22.5	8	14	0.25	5	1	97	23.5	30.25	26	3	198	16.5	12	20	6.5	5.5	42	131	2.5	29	8.5
1/19/2014	1125	21	51	9	25.5	44	16	28	16	30		9	0.25	5		85.5	19.5	18.25	16	3	160	12.5	12	16	4.5	5.5	30	139	0.5	24	8.5
1/26/2014	1125	21.5	52	9	32.8	44.5	26	36	15	5	8	15	4.25	15		89.5	18	29.25	26	3	156	16.5	12	14	4.5	8.5	30	98	2.5	26	8.5
2/2/2014	1125	3.5	76	9	15.9	22.5	41	75		5	9	173	6.5	34	52	92.5	19	29.6	25.6	3	156	48	12	14	5.25	10.5	39		6	162	8.5
2/9/2014	7.5	6.5	28.5	51	7.6	26	6	37.5	40	13	4	3.7	3	37	87	60	12.5	21.4	4.4	4.7	151	47	*48*	54.5	9.5	6.5	131		6.5	178	5
2/16/2014	5	3.5	13.3	10	2.5	9.5		11	17	4	17.5	7.5	6	12	34	45	5.5	8	1.7		82		8	37	6.25	27.5	10		5	114	3
3/2/2014	11.5	35	78	10.5	21	52.75	26	87	36	17.5		20.75	2.5	28	37	92	18	23.5	31	3	78	24	12	15	5.25	5.5	77	113	6	38	8.5
3/9/2014	5.25	27	20	16	40.2	62.75	30	4735	24.5	7.2		4.25	5	18.4	50	15	7.5	36.75	10	6	45.5			25.5	11	2	110	133.75		37.5	0.6
3/16/2014	3.55	37	22	6	28.3	158.25	64.4	50.75	34.5	12.8		4.25	7	10.4	50	22	32.7	29.55	12.5	6	103		5	36.5	46		100	50.75		20	0.6
3/23/2014	14	18	13	6	32.4	40	49		107	29.6		7.5	7	13	50	37	15.5	43.5	47	4.75	65	2.5		26	4.5	7.5	151	95.9		48	5
3/30/2014	14	18	13	6	25.6	49	49		89	23.2		7.5	7	13	50	37	14.5	40.5	34	3.75	65	5		25	8	7.5	149	99.9		58	5
4/6/2014	2.5		25	30	4			15	20	15.75				40	20	6		15	23		200	5		30		5	32	58		38	
4/13/2014	125	2	31.5	21.25	28	22.15	18.8	15	35.6	18.15		4	1.2	29.5	19.5	11	9.25	23.4	25.5	7.7	140		3	23.15	9.25		23	53.6		26.5	16.2
4/20/2014	3.5	2.4	22	10	40.25	79.3	6.8	42	64.2	33.45				9.6	35	16.5	3.25	50.75	24	3.25	319	30		24.9	9.95	1.75	85	138.1	1.5	54.5	
4/27/2014	14	18	13	6	25.6	20	24		71.15	23.2		10	7	13	50	34	14.5	40.5	34		273	125		25	8	7.5	149	71.1		67	5
5/18/2014	12	23	17	36.25	2735	30.6		65.75	93			2.5		38.4	45	46	7.5	57.8	33	6.75	378	91.5		32.5	3.5	8	88	126.65		64.5	0.6
5/25/2014		14	12	16.5	20.35	23.6		39.8	61.2					13	13	16.5	4.5	36	33.5	4.25	319	75		3.15	11	3	75	67.2	3	34	3
6/1/2014	12.75	66	39	26.5	32.2	46	100.75		53.5	23.5		5		81.4	31	62	9.5	35	52	6	441	13		34.25	14.75	8	108	165.75	5.75	68.5	4.6
6/8/2014	12.5	15	14	6	19.65	43	33.4		160	18.15		15.5	28	50	18	15.7	40.5	36.5	3.75	361	70	28		7	2.5	158	106.9		42.5		
6/15/2014	4.5	7	17	36.25	17.25	78.6	47		75	11.6		2.5		30	45	40		36.2	33	0.75	360	9.5		25	3.5	5	63	107.9		52	
6/22/2014	13.25	96	24	15	36.45	197	77.5		52	13.65		9	7.75	39.5	68	93	6.25	52.5	11	3.25	322	16.75		28.5	1	0	82	76.5	10	60.5	5
6/29/2014	13.25	96	24	15	36.45	190.75	77.5		52	13.65		9	7.75	39.5	68	93	6.25	52.5	11	3.25	358	16.75		28.5	15	0	82	76.5	10	60.5	5

**Table 4:** FJespacial data source.

Feria	Brócoli	Cebolla blanca	Tomate	Tomate de arbol	Zanahoria	*x*	*y*
Colta	6	60	26	30	30	−78.723827	−1.8880031
Tisaleo	37.9	36	88.25	185	47.5	−78.6922532	−1.4095144
Riobamba1	8	9.6	18	0	0	−78.6736758	−1.6615342
Riobamba2	26	42.5	60	15	17.5	−78.6729892	−1.6608907
Riobamba3	0	10	8	8	5	−78.6686976	−1.6602473
Riobamba4	2.5	74	60	35	29.5	−78.6588056	−1.6762586
Cevallos	9	9.6	51	45	20	−78.6566384	−1.2571434
Riobamba5	7.2	50	30	35	20	−78.655823	−1.6871008
Riobamba6	6	65	42	27	29	−78.6538275	−1.6759905
Riobamba7	2	0	10	0	5	−78.6500294	−1.6780281
Riobamba8	6	26.5	33	0	36	−78.6497031	−1.6746817
Riobamba9	2.5	0	6.9	0	3	−78.6462744	−1.6894172
Chambo	21	50	30	20	20	−78.6076928	−1.7302981
Pillaro	20.1	141.5	92	78	40	−78.551033	−1.3283574

## Data Availability

The data used are provided by the General Coordination of Marketing Networks of the Ministry of Agriculture and Livestock of Ecuador, within the framework of an interinstitutional agreement with the Salesian Polytechnic University.
